# Improvement of Hypertriglyceridemia by Roasted *Nelumbinis folium* in High Fat/High Cholesterol Diet Rat Model

**DOI:** 10.3390/nu12123859

**Published:** 2020-12-17

**Authors:** Hye Yoom Kim, Mi Hyeon Hong, Kwan Woo Kim, Jung Joo Yoon, Jung Eun Lee, Dae Gill Kang, Ho Sub Lee

**Affiliations:** 1 Hanbang Cardio-Renal Research Center & Professional Graduate School of Oriental Medicine, Wonkwang University, Iksan 54538, Korea; hyeyoomc@naver.com (H.Y.K.); mihyeon123@naver.com (M.H.H.); swamp1@naver.com (K.W.K.); mora16@naver.com (J.J.Y.); 2 Department of Physiology, College of Oriental Medicine, Wonkwang University, Iksan 54538, Korea; 3 Clinical Medicine Division, Korea Institute of Oriental Medicine, Daejeon 34054, Korea; steineun@kiom.re.kr

**Keywords:** roasted *Nelumbinis folium*, high fat/cholesterol diet, hypertriglyceridemia, metabolic syndrome, obesity

## Abstract

Hypertriglyceridemia is a condition characterized by high triglyceride levels and is a major risk factor for the development of cardiovascular diseases. The present study was designed to investigate the inhibitory effect of roasted *Nelumbinis folium* (RN), which is a medicinal substance produced by heating lotus leaves, on lipid metabolism in high fat/cholesterol (HFC) diet-induced hypertriglyceridemia. Except for those in the control group, Sprague–Dawley rats were fed an HFC diet for four weeks to induce hypertriglyceridemia. During the next nine weeks, the control, regular diet; HFC, HFC diet, FLU, fluvastatin (3 mg/kg/day); RNL, RN (100 mg/kg/day); RNH, RN (200 mg/kg/day) were orally administered together with the diet, and the experiments were conducted for a total of 13 weeks. The weight of the epididymal adipose tissue, liver, and heart of rats in the HFC diet group significantly increased compared to those in the control group but improved in the RN-treated group. It was also confirmed that vascular function, which is damaged by an HFC diet, was improved after RN treatment. The levels of insulin, glucose, triglycerides, total cholesterol, and low-density lipoprotein increased in the HFC diet group compared to those in the control group, while the administration of RN attenuated these parameters. In addition, the administration of RN significantly reduced the gene expression of both LXR and SREBP-1, which indicated the inhibitory effect of the biosynthesis of triglycerides caused by RN. The results indicated that RN administration resulted in an improvement in the overall lipid metabolism and a decrease in the concentration of triglycerides in the HFC diet-induced rat model of hypertriglyceridemia. Therefore, our findings suggest that the RN can be a candidate material to provide a new direction for treating hypertriglyceridemia.

## 1. Introduction

An excessive intake of carbohydrates and animal fat increases the development of cardiovascular diseases [1]. Moreover, the risk of these diseases could be increased by hyperlipidemia, high blood pressure, diabetes, obesity, family history, smoking, stress, and lifestyle preferences. In particular, hyperlipidemia is the most important independent factor of coronary artery disease [2,3]. According to previously published reports, cancer, heart disease, cerebrovascular disease, ischemic heart disease, and cerebrovascular disease are associated with a high risk of mortality [4]. Hyperlipidemia and hypertriglyceridemia induced by an excessive intake of fat, sugar, saturated fatty acids, and cholesterol are associated with mortality [3,4]. Hyperlipidemia and hypertriglyceridemia are associated with high blood triglyceride (TG) and high low-density lipoprotein (LDL) levels [5,6]. It is not yet known whether hyperlipidemia and hypertriglyceridemia are the primary causes of coronary diseases, but it is known that these diseases constitute major risk factors for the development of these disorders [5]. Restricting fat and cholesterol in patients with hypercholesteremia for six months resulted in significantly decreased LDL cholesterol levels in the blood and the decrease of arteriosclerosis, indicating that the intake of fat and cholesterol affects the development of hyperlipidemia and hypertriglyceridemia [7]. Fat is required to absorb fat-soluble vitamins or essential fatty acids, which serve as high-energy sources. However, excessive fat is converted to intestinal dietary cholesterol and chylomicrons, which are composed of 82% of TGs. The chylomicrons are transported to fat tissues, where they are stored primarily in the form of neutral lipids [8]. TGs play an essential role as a source of energy, such as for the muscles, and they are stored in the adipose tissue [9]. Obesity can result if the storage of TGs increases by more than a certain amount through an excessive ingestion of animal fat, which leads to an increased risk and higher incidence of cardiovascular diseases, such as myocardial infarction and arteriosclerosis [10]. Therefore, hyperlipidemia and hypertriglyceridemia are risk factors for arteriosclerosis and coronary artery diseases [11,12]. In this regard, this investigation sought to evaluate whether the extract of roasted Nelumbinis folium (RN) could reduce hypertriglyceridemia.

*Nelumbinis folium* (RN) is the lotus leaf, which is known to have the following effects: cooling of heat, smoothing of the metabolism of water in the body, clearing the blood, and removing the extravasated blood [13,14,15]. Roasted RN is a medicinal substance produced by folding the lotus leaf, placing it in a pot, sealing it with salt-rich mud, pressing it with a heavy object, and heating it to a high temperature. This preparation is known to be used for various hemorrhagic diseases and postpartum bleeding and dizziness owing to its enhanced astringing effect, blood stasis removal, and stopping of bleeding [16]. This medicinal preparation is expected to increase the effect of the lotus leaf, but it has not been investigated yet. Therefore, this study was conducted to examine whether the administration of RN could improve metabolic diseases, such as obesity and insulin resistance, caused by high-fat/high-cholesterol diets in rats.

## 2. Materials and Methods

### 2.1. Preparation of Roasted Nelumbinis folium (RN) Component Analysis

The leaf of roasted *Nelumbinis folium* (RN) herb was purchased from the Herbal Medicine Cooperative Association, Iksan, Jeonbuk, South Korea. The RN (100 g) were boiled with 1 L of distilled water at 100 °C for 2 h. The extract was filtered and centrifuged at 3000 rpm for 20 min at 4 °C. The supernatant was concentrated, and then the RN extract was lyophilized with a freeze dryer. The extract was dried (extract yield: 10.1%) and stored at −70 °C. The chemical components of RN were analyzed using ultra-performance liquid chromatography equipped with quadrupole Q exactive Orbitrap mass spectrometry UPLC/QE Orbitrap MS. Details about the UPLC/QE Orbitrap MS analysis of compounds in RN are described in the Materials 1. Additionally, details about identified compounds are listed in Table S1. The extract voucher specimen (No. HBA 162-01) is deposited in the herbarium of the Hanbang Cardio-renal Research Center, Wonkwang University.

### 2.2. Animals and Treatment

All experiments were performed according to protocols that were approved by the Animal Care Committee of Wonkwang University and by the Institution Animal Care and Use Committee (IACUC) Certification of the Wonkwang University, Korea (WKU16-95). Male Sprague–Dawley (SD) rats (8-week-old) were purchased from Samtako (Samtako Bio Korea Inc., Osan, Korea) and used for a total of 13 weeks. The animals were housed in a temperature-controlled environment and maintained on a 12 h dark/12 h light cycle at a temperature of 23 °C ± 2 °C and relative humidity of 50–60% with free access to laboratory food and water. After a week of adaptation, the SD rats were randomly divided into the following five groups with fifteen rats per group: Control, regular diet (*n* = 12); HFC, HFC diet (*n* = 12); FLU, fluvastatin (3 mg/kg/day, *n* = 10) with HFC diet; RNL, RN (100 mg/kg/day, *n* = 10) with HFC diet; RNH, RN (200 mg/kg/day, *n* = 10) with HFC diet. The control group was fed a regular diet for 13 weeks. The HFC diet group was fed a high-fat, high-cholesterol diet, of which 60% (kcal/kcal) was derived from fat and 22.5% (kcal/kcal) from lard (cat no. D12492, Research Diets, Inc., New Brunswick, NJ, USA). All groups were fed an HFC diet for four weeks, whereas FLU, RNL, and RNH groups were provided an HFC diet for the next nine weeks. Fluvastatin was used as a positive control in this study, since it is a drug used for hyperlipidemia, which acts by reducing cholesterol and TG levels in plasma and decreases fat accumulation in tissues.

### 2.3. Changes in Vascular Tone Relaxation and Systolic Blood Pressure

Non-invasive blood pressure was measured in all animal groups using the tail cuff CODATM High Throughput System (Kent Scientific Corporation, Torrington, CT, USA). The aortic ring was prepared as previously reported, and a vascular relaxation experiment was performed [17]. Changes in the isobaric tension in the thoracic aorta were recorded using a transducer (Grass FT 03, Grass Instrument Co, MA, USA) connected to a Grass Polygraph recording system (Model 7E, Grass Instrument Co, Quincy, MA, USA). After inducing contraction with phenylephrine (1 μM), treatment with acetylcholine (0.3 nM to 3 μM) was performed in a dose-dependent manner to confirm its relaxation effect.

### 2.4. Hematological Analysis

The blood was collected 13 weeks later as the experiment was completed, and the plasma was obtained via centrifugation at 13,000 rpm for 10 min. Plasma hematological tests to measure the total cholesterol, TGs, glucose, and HDL were performed using an automated clinical chemistry analyzer (FUJI DRI-CHEM NX700, FUJIFILM Corporation, Tokyo, Japan). The levels of fatty acids (ab65341, Abcam Inc., Bristol, UK), fatty acid synthase (CSB-E16440r, Cusabio Biotech Co, Houston, TX, USA), and serum insulin concentration (EIA-2943, DRG Ultra-Sensitive Rat Insulin ELISA, DRG International Inc., Springfield, NJ, USA) were determined using commercially available ELISA kits.

### 2.5. Extraction of RNA and Quantitative Real-Time Polymerase Chain Reaction (qPCR) Analysis

The total RNA of the liver tissue was extracted using TRIzol reagent (Ambion, Carlsbad, CA, USA), and the cDNA was synthesized using a cDNA kit (Applied Biosystems, Waltham, MA, USA) according to the manufacturer’s recommendations. The samples were analyzed using SYBR Green PCR Master Mix (Applied Biosystems) through a quantitative real-time polymerase chain reaction. The sequences of primers used in this experiment were as followed: LXR-1, sense: 5′-GAAGAAACTGAAGCCGCAAG-3′, anti-sense: 5′-TAGCATCCGTGG3AACATCA-3′; SREBP-1c, sense: 5′-GGTTTTCAACGACAT CGAAGA-3′, anti-sense: 5′-CGGGAAGTC ACT3TCTTGGT-3′; GAPDH, sense: 50-CGA GAA TGG GAA GCT TGT CAT C-30, anti-sense: 50-CGG CCT CAC CCC ATT TG-30. The PCR was started at 95 °C for 15 min (hot start) to activate the AmpliTaq polymerase, followed by a 45-cycle amplification; denaturation at 94 °C for 20 s, annealing at 60 °C for 30 s, extension at 72 °C for 60 s, and plate reading at 60 °C for 10 s. The temperature of PCR products was elevated from 65 to 95 °C at a rate of 0.2 °C/1 s, and the analysis of gene expression was conducted by Step One Plus Real-Time PCR system (Applied Biosystems).

### 2.6. Concentration of Hepatic TGs

Liver tissue was homogenized in phosphate-buffered saline, and lipids were extracted using a chloroform–methanol (2:1) mixture. Liver TG levels in 50 mg of liver tissue were determined according to the method by Bligh and Dyer [18] and quantified using an enzymatic assay (Roche Diagnostics, Rotkreuz, Switzerland).

### 2.7. Histopathological Staining of Tissues

The collected thoracic aorta tissues were fixed for three days in 10% formalin (pH 7.4) and washed with water to remove the remaining formalin from tissues. The paraffin blocks were sectioned in 6–7-μm slices using a microtome (Thermo Electron Corporation, Pittsburg, PA, USA).

Liver and epididymal fat tissues were fixed by immersion in 4% paraformaldehyde for two days at 4 °C and then incubated with 30% sucrose for two days. The frozen sections were cut using a Shandon Cryotome SME (Thermo Electron Corporation, Pittsburg, PA, USA). The slides were stained with hematoxylin and eosin (H&E) and oil red O staining for histopathological studies and observed using light microscopy (EVOSTM M5000, Thermo Fisher Scientific, Bothell, WA, USA).

### 2.8. Statistical Analysis

The data are expressed as mean ± standard error. Differences between the mean values were analyzed using the Student’s *t*-test by using SigmaPlot version 10. Differences between groups were considered significant at *p* < 0.05.

## 3. Results

### 3.1. Analysis of the Components of Roasted RN

**Figure 1:**
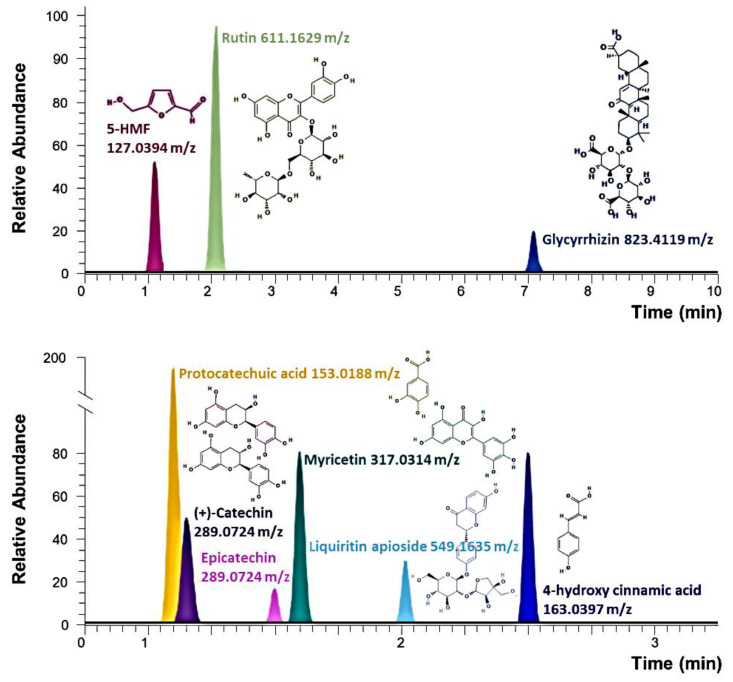
Analysis of the components of roasted *Nelumbinis folium* (RN). Extracted ion chromatograms (EIC) of UPLC/QE Orbitrap MS analysis of the positive (up) and negative (down) charged molecular ion shows nine major compounds.

The known components of RN were verified by comparison with 9 pure compounds, namely, (+)-catechin, 4-hydroxycinnamic acid, 5-hydroxymethylfurfural, epicatechin, glycyrrhizin, liquiritin apioside, myricetin, protocatechuic acid, and rutin. Based on the retention time, mass spectroscopy, and MS/MS fragmentation patterns, among others, six components of RN were tentatively identified as adenosine, isoquercitrin, quercetin-4′-glucoside, threonic acid, trigonelline, and valine (Figure 1). In particular, the protocatechuic acid and rutin has been identified as a major bioactive compound of the RN.

### 3.2. Effect of RN on Body and Tissue Weight Changes in High Fat/Cholesterol Diet Fed Rats

The effects of RN on the body weight were compared between groups after providing a regular diet and a high fat/cholesterol (HFC) diet for 10 weeks. The body weight at the beginning of the experiment was not significantly different among the groups. The weight at the end of the experiment showed a significant increase of 10.9% (*p* < 0.05) in rats in the group fed an HFC diet compared to those in the control group, but a significant decrease of 7.1% (*p* < 0.05) was observed in the rats in the RN group. The weight of epididymal adipose tissue, liver, and heart of rats in the HFC diet group was significantly increased compared to that in the control group but was decreased in the RN-treated group. There was no significant difference in the weight of the kidneys among groups (Table 1).

### 3.3. Effects of RN on Vascular Dysfunction in HFC Diet Rats

**Figure 2:**
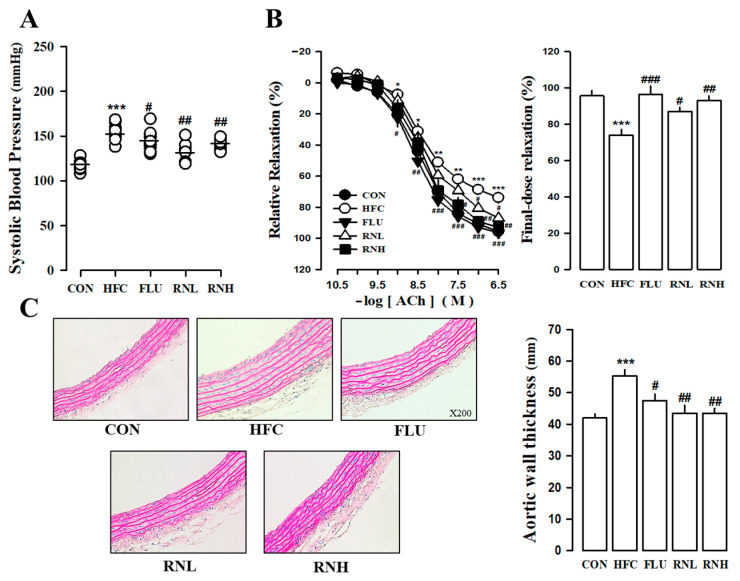
Systolic blood pressure variation in high fat/high cholesterol (HFC) diet rats for analysis of *Nelumbo nucifera* (RN)-treated cardiovascular functional characteristics (**A**). Effect of RN on vascular relaxation by acetylcholine (Ach) in the thoracic aortic ring and on the relaxation rate at the final acetylcholine dose (**B**). Representative histological images of hematoxylin and eosin (H&E) staining and aortic media thickness in the thoracic aorta (**C**). CON, regular diet; HFC, HFC diet; FLU, fluvastatin (3 mg/kg/day) with HFC diet; RNN, RN (100 mg/kg/day) with HFC diet; RNH, RN (200 mg/kg/day) with HFC diet. The values represent the means ± standard error. * *p* < 0.05, and ** *p* < 0.01, and *** *p* < 0.001 vs. CON; # *p* < 0.05, ## *p* < 0.01, and ### *p* < 0.001 vs. HFC.

After 11 weeks of dietary intervention, the systolic blood pressure was found to be significantly higher in rats in the HFC diet-fed group than those in the control group. On the other hand, the RN-treated HFC diet rats had a significantly lower systolic blood pressure than those in the HFC diet group (Figure 2A). The improvement in vascular reactivity by RN in the HFC diet-fed rats was confirmed using isolated aortic rings. Our findings indicated that acetylcholine-induced endothelial-dependent vascular relaxation, which was significantly weakened in HFC diet rats (73.89 ± 3.46%, *p* < 0.001 vs. CON), significantly recovered after RN treatment (Figure 2B). The vessel thickness was measured using hematoxylin and eosin staining to evaluate the effect of vascular morphological changes. The thoracic aortic tissue showed a thickening of the blood vessels in the HFC diet rats, which was improved by RN treatment (Figure 2C). These results suggested that RN administration could ameliorate vascular dysfunction in rats fed an HFC diet.

### 3.4. Effect of RN on Hematological Changes in HFC Diet-Fed Rats

**Figure 3:**
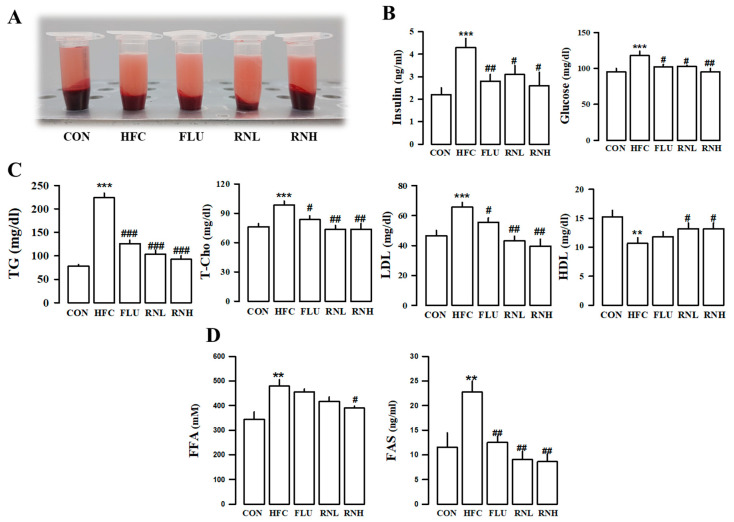
Hematological changes in the treatment of roasted *Nelumbo nucifera* (RN) in high fat/cholesterol (HFC) diet Rats. Representative plasma images of HFC diet-fed rats appear turbid compared to those of the control group (**A**). Representative plasma image of HFC diet rats that appear turbid compared to the control group (**A**). Measurements of plasma insulin and glucose (**B**) TG, T-Cho, LDL, and HDL (**C**); FFA and FAS (**D**). CON, regular diet; HFC, HFC diet; FLU, fluvastatin (3 mg/kg/day) with HFC diet; RNN, RN (100 mg/kg/day) with HFC diet; RNH, RN (200 mg/kg/day) with HFC diet; TG, triglyceride; T-Cho, total cholesterol; LDL, low-density lipoprotein; HDL, high-density lipoprotein; FFA, free fatty acid; FAS, fatty acid synthase. The values represent the means ± standard error. ** *p*<0.01 and *** *p* < 0.001 vs. CON; # *p* < 0.05, ## *p* < 0.01, and ### *p* < 0.001 vs. HFC.

The levels of insulin, glucose, TG, total cholesterol (T-Cho), and low-density lipoprotein (LDL) were higher in the HFC diet group compared to those in the control group. However, the administration of RN decreased these responses (Figure 3C). Moreover, high-density lipoprotein (HDL) levels were significantly increased in the group administered RN (Figure 3C). In addition, fluvastatin lowered total cholesterol, LDL cholesterol, and triglycerides in the blood, and RN treatment was as effective as a known positive control. TGs are converted into free fatty acids (FFAs) through hydrolysis and are secreted in the blood, which is then transported to the muscles or liver. Therefore, the levels of FFAs and fatty acid synthase (FAS) were measured (Figure 3D). Rats in the HFC diet-fed group were found to have increased levels of FAS and FFAs compared to those in the regular diet group, while those in the RN treatment group had decreased levels, which showed an improvement in TG production in the blood.

### 3.5. Effect of RN on Changes in the Size of Epididymal Adipocytes in HFC Diet Rats

**Figure 4:**
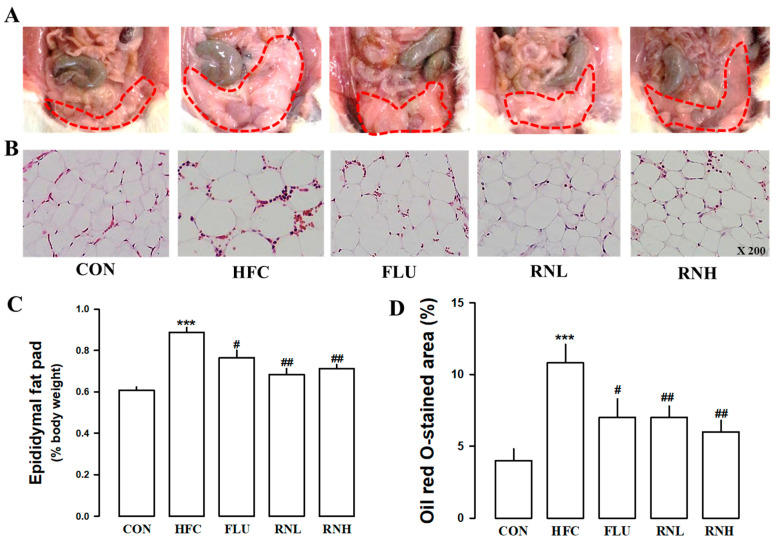
Effect of roasted *Nelumbo nucifera* (RN) treatment on the changes in the epididymal fat pad on high fat/high cholesterol (HFC) diet rat. Representative image of the visceral fat distribution of each group (**A**). The red line indicated the degree of visceral fat distribution. Representative microscopic photograph of an epididymal fat pad using the oil red O staining method (**B**). Lower panel indicated the weight (**C**) and size (**D**) of adipose cells. CON, regular diet; HFC, HFC diet; FLU, fluvastatin (3 mg/kg/day) with HFC diet; RNN, RN (100 mg/kg/day) with HFC diet; RNH, RN (200 mg/kg/day) with HFC diet. The values represent the means ± standard error. *** *p* < 0.001 vs. CON; # *p* < 0.05 and ## *p* < 0.01 vs. HFC.

The abdominal fat areas were marked with red lines and compared by groups, the areas of abdominal fat increased in HFC diet rats, and treatment of RN reduced the abdominal fat area (Figure 4A). RN administration significantly reduced the weight of epididymal adipose tissue in HFC diet-fed rats (Figure 4C). Therefore, to observe changes in the size of the adipocytes, oil red O staining of epididymal adipose tissue was performed (Figure 4B,D). Therefore, it was confirmed that the size and amount of fat increased by the HFC diet were reduced by RN treatment.

### 3.6. Effect of RN on Liver Function and Hepatic Lipids in HFC Diet-Fed Rats

**Figure 5:**
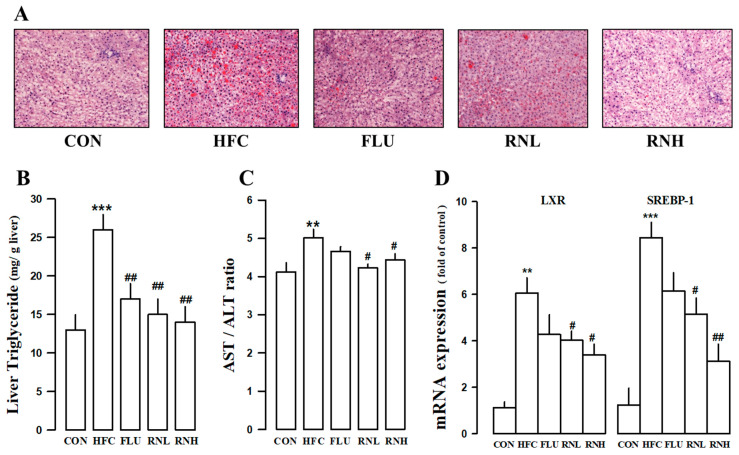
Effects of roasted *Nelumbo nucifera* (RN) on hepatic histopathology and function in rats fed a high fat/cholesterol (HFC) diet. Histopathological changes were identified within the liver tissue using hematoxylin and eosin staining (**A**). Graph of hepatic triglyceride levels (**B**), AST/ALT ratio in plasma (**C**), and hepatic *LXR* and *SREBP-1* gene expression levels (**D**). CON, regular diet; HFC, HFC diet; FLU, fluvastatin (3 mg/kg/day) with HFC diet; RNN, RN (100 mg/kg/day) with HFC diet; RNH, RN (200 mg/kg/day) with HFC diet; LXR, liver X receptor; SREBP-1c, Sterol regulatory element-binding protein-1c; AST, aspartate aminotransferase; ALT, alanine aminotransferse. The values represent the means ± standard error. ** *p* < 0.01 and *** *p* < 0.001 vs. CON; # *p* < 0.05 and ## *p* < 0.01 vs. HFC.

To investigate the accumulation of fat in the liver of the rats in each group, sections of the liver were prepared using the frozen samples, and oil red O staining was performed. The results indicated that liver lipids were detected in HFC diet-fed rats, and the group treated with RN showed a decrease (*p* < 0.01) in lipid distribution in the liver compared to the HFC diet-fed group (Figure 5A). In particular, treatment with RNH more effectively suppressed lipid accumulation. A similar trend was observed in liver TG levels (Figure 5B). The aspartate aminotransferase (AST)/alanine aminotransferse (ALT) ratio is an indicator of hepatic cell damage and is frequently used to determine liver function. The effect on the reduction of the AST/ALT ratio was significant in the RNL group, while the decrease in the FLU group (*p*-value of 0.0581) was not significant (Figure 5C). To investigate the role of genes regulating the synthesis of triglycerides in HFC diet rats, real-time PCR was conducted using liver-tissue samples. HFC diet induced the expression of *LXR* and *SREBP-1*, which are associated with lipogenesis, biosynthesis of fatty acids, and the maturation of triglycerides. The administration of RN significantly reduced the gene expression of both *LXR* and *SREBP-1*. In FLU groups, LXR (*p*-value of 0.060) and SREBP-1 (*p*-value of 0.054) showed a decrease in gene expression levels, but it was not significant (Figure 5D), which indicated the inhibitory effect of RN on the biosynthesis of triglycerides. These results suggested that the RN could regulate TG levels.

## 4. Discussion

Various studies have reported that HFC diet-fed animals develop abnormal lipid metabolism, hypertriglyceridemia, obesity, glucose tolerance disorders, metabolic syndrome, atherogenic dyslipidemia, and cardiovascular diseases [19,20,21]. In this study, rats that consumed an HFC diet developed hypertriglyceridemia. Moreover, the current investigation sought to verify whether roasted RN could alleviate or reverse hypertriglyceridemia induced by an HFC diet in rats.

Previous studies indicate that RN has anti-insomnia, anti-anxiety, anti-melanin production [22,23], antidepressant [24], anti-obesity, antioxidant, anti-cardiovascular disease, and anti-cancer effects [25,26]. Lotus leaf is a traditional medicinal ingredient that has been used for hundreds of years and is widely used as medicines and food. In the case of RN, there is only an example of its production, but its efficacy is still unknown. We compared the relaxation effect of the roasted versus unroasted RN and confirmed that roasted RN had a better effect; then, we examined its effect by selecting an RN (Figure S1). Fluvastatin, a drug used in the management of hyperlipidemia, was used as a positive control to reduce cholesterol, neutral fats in the plasma, and lipid accumulation in the liver [27]. The difference in organ and fat weight between animals treated with HFC and those untreated is one of the most important pieces of evidence obtained from morphological comparisons [28]. Additionally, the weight of organs and fat, especially the liver, is an essential factor in understanding obesity caused by an HFC diet [29]. The HFC diet resulted in an increase in the weight of organs, especially the liver, and was confirmed to be reversed after RN administration. Moreover, an HFC diet is known to cause obesity, and the size of fat cells also increases significantly [30,31]. Observation of abdominal fat resulted in a significant decrease in the distribution of abdominal fat, which had been increased by the HFC diet, and by reducing epididymal fat pad, the anti-obesity effect of RN could be confirmed.

Triglycerides (TG) are a major energy source, except for the brain and red blood cells. The liver plays an important role in the synthesis and transport of TGs. However, there are more problems than benefits if TGs are produced in excess [32]. In addition, the in vivo lipid imbalance caused by HFC diets increases the concentration of plasma lipoprotein LDL cholesterol and reduces the HDL cholesterol levels, leading to cardiovascular diseases, such as hypertension and arteriosclerosis [33,34]. Therefore, in hypertriglyceridemia induced by an HFC diet, RN intake can be useful in the treatment of cardiovascular diseases, as it can decrease TG, LDL, and HDL levels [32,33]. The TGs produce free fatty acids (FFAs) through hydrolysis reactions and are secreted into the blood, which move to the muscles or liver [35]. Plasma FFA levels are controlled by FFA distribution between the plasma and adipose tissues and are mediated by lipoprotein lipase and hormone-sensitive lipase [36]. The induced FFAs increase neutral fat synthesis in the liver as well as the secretion of very-low-density lipoprotein (VLDL) [37,38]. Therefore, the levels of FAS and FFAs in the blood were measured and were found to be increased in the rats fed an HFC diet compared to those fed a regular diet; RN could reverse this effect. These findings suggest that RN plays an important role in regulating plasma FFA levels.

Hypertriglyceridemia is an independent risk factor for coronary artery disease [39,40] and is known to contribute to arteriosclerosis and coronary artery disease development [41]. Moreover, several studies have demonstrated that hypertriglyceridemia can impair endothelial function and thus can affect blood pressure and vasodilation [42,43]. In addition, systolic dysfunction is reported to be caused by dietary metabolic syndrome and increased contractile dysfunction [44]. Vascular dilations in the blood vessels induced by acetylcholine have proved that the endothelium is damaged as a result of the HFC diet, thereby bringing about fluctuations in blood pressure. On the other hand, the reduction in aortic ring relaxation and increase in blood pressure due to acetylcholine were significantly improved by treatment with RN. Therefore, RN is considered to improve vascular dysfunction. In the case of obesity, when fat accumulates in hepatocytes owing to dyslipidemia, liver weight increases and the entire liver becomes enlarged, resulting in further liver damage [45,46]. In particular, treatment with RN effectively suppressed lipid accumulation and lowered liver TGs.

Since the synthesis and breakdown of TGs in hypertriglyceridemia occurs in the liver, it is important to check the levels of liver enzymes, including ALT and AST, to determine the degree of liver damage. Hypertriglyceridemia is correlated with abnormal liver function and indicators, such as ALT and AST, can be used to determine the extent of liver damage [47]. Results from this study confirmed that RN improved the ALT/AST ratio in HFC diet rats. In hypertriglyceridemia induced by the HFC diet, the RN administration decreased the concentration of ALT and AST, indicating that the HFC diet caused severe damage to the hepatic tissue and that the administration of RN could alleviate liver damage caused by hypertriglyceridemia.

**Figure 6:**
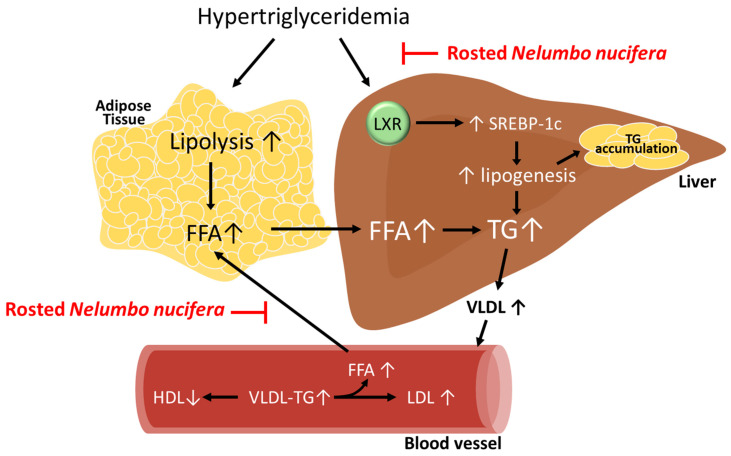
Schematic diagram of the effect of roasted *Nelumbo nucifera* (RN) in improving hypertriglyceridemia. TG, triglyceride; FFA, free fatty acid; LXR, liver X receptor; SREBP-1c, Sterol regulatory element-binding protein-1c; VLDL, very-low-density lipoprotein; HDL, high-density lipoprotein; LDL, low-density lipoprotein.

Additionally, in this study, the liver and blood were examined to determine whether RN affected the accumulation and synthesis of TGs following the HFC diet. *LXR*α and *SREBP-1* are transcription factors that play an important role in the synthesis of TGs in the liver. LXR activation increases TG synthesis and hepatic TG secretion [48,49]. Moreover, the hepatic regulation of SREBP-1c in fatty acid synthesis leads to an increase in TG levels and promotes VLDL production [50,51]. Following an HFC diet, LXR increases the expression of SREBP-1, which is a precursor of fatty acids and TGs, resulting in the accumulation of TGs in the liver tissue [52]. The administration of RN inhibited the accumulation of TGs, indicating its efficacy in reversing hypertriglyceridemia. Figure 6 shows a schematic image of RN treatment for improving liver dysfunction in hypertriglyceridemia.

However, it is necessary to acknowledge and resolve the two major limitations of current research. First, unfortunately, separate chemical compounds, such as protocatechuic acid and rutin, have been analyzed for RN, but related studies have not been conducted. Second, our results showed that the hypertriglyceridemia improvement effect of RN was not concentration-dependent and could not be clearly explained, because it was at a similar level or showed only a slight difference in high dosage. Therefore, further studies are necessary to confirm the effect of RN concentration-specific and chemical compounds.

## 5. Conclusions

In this study, the administration of RN resulted in an improvement in overall lipid metabolism and a decrease in the concentration of triglycerides in a rat model of HFC diet-induced hypertriglyceridemia. In particular, inhibiting the production of TGs by RN was found to be related to the inhibition of triglyceride synthesis by inhibiting LXR activity. Therefore, the results of our study suggest that RN has the potential to be used in the treatment of hypertriglyceridemia.

## Figures and Tables

**Table 1:** Effect of roasted *Nelumbo nucifera* (RN) on the change of tissue weight in high fat/cholesterol (HFC) diet rats.

Weight (g)	CON	HFC	FLU	RNL	RNH
Final Body	449.41 ± 7.67	498.13 ± 6.88 ***	476.53 ± 4.43 ^#^	466.39 ± 5.44 ^#^	488.60 ± 9.26
Epididymal fat pad	2.89 ± 0.08	4.44 ± 0.11 ***	3.89 ± 0.16 ^#^	3.41 ± 0.17 ^###^	3.69 ± 0.16 ^##^
Liver	9.37 ± 0.64	11.01 ± 0.36 ***	8.81 ± 0.49 ^##^	9.11 ± 0.46 ^##^	9.39 ± 0.33 ^##^
Heart	1.34 ± 0.02	1.44 ± 0.02 *	1.41 ± 0.06	1.39 ± 0.04 ^#^	1.37 ± 0.05 ^#^
Kidney	2.46 ± 0.03	2.59 ± 0.06	2.44 ± 0.06	2.46 ± 0.04	2.36 ± 0.03

CON, regular diet; HFC, HFC diet; FLU, Fluvastatin (3 mg/kg/day) with HFC diet; RNN, RN(100 mg/kg/day) with HFC diet; RNH, RN(200 mg/kg/day) with HFC diet. The values represent the means ± standard error. * *p* < 0.05 and *** *p* < 0.001 vs. CON; ^#^ *p* < 0.05, ^##^ *p* < 0.01, and ^###^ *p* < 0.001 vs. HFC.

## Supplementary Materials

The following are available online at https://www.mdpi.com/2072-6643/12/12/3859/s1, Figure S1: Dose-dependent vascular relaxation effect of roasted and non-roasted *Nelumbinis folium* in thoracic aortic rings; Materials 1. UPLC/QE Orbitrap MS analysis of Compounds in roasted *Nelumbinis folium* (RN); Table S1. List of identified compounds in roasted *Nelumbinis folium* (RN).
