# Supplementary material for: Improvement of Hypertriglyceridemia by Roasted Nelumbinis folium in High Fat/High Cholesterol Diet Rat Model
Source: Nutrients. 2020 Dec 17;12(12):3859. doi: 10.3390/nu12123859 (PMC7766402; doi:10.3390/nu12123859)
Supplement: Supplementary file 1 [file nutrients-12-03859-s001.pdf]

nutrients

MDPI

*Supplementary method*

## 1. UPLC/QE Orbitrap MS analysis of Compounds in roasted Nelumbinis Folium (RN).

A 0.10g of RN was prepared in 1.5 ml e-tube and added 750 ul of 70% methanol (MeOH) for solvent extraction. After centrifugation at 13,200 rpm for 10 minutes at 4°C, 500 ul of supernatant was collected in fresh 1.5 ml e-tube. Briefly, 100 ul aliquots of this solution were diluted with 70% acetonitrile (ACN) until 10ppm for ingredients analysis. The UPLC/QE Orbitrap MS which was equipped with a heated electrospray ionization (HESI) source was used. Chromatographic separation were performed on a Acquity UPLC CSH™ C18 (2.1 x 100 mm, 1.7 um, Waters, USA) column, using binary gradient system. The mobile phase comprised 0.1% formic acid (FA) in distilled water (DW, solvent A) and ACN (solvent B) and elution gradient was follows; 20 to 60% B; 0-8 min→60 to 90% B; 8-10 min→90 to 98% B; 10-11 min and hold 1 min→98 to 20% B; 12-13 min→equilibration until 20 min. Separation were accomplished at a column flow of 0.35 ml/min and temperature of 40°C. The injection volume of samples were 5 ul and autosampler were kept at 5°C. All analyzes were performed in both ionization modes (negative and positive) based on Full MS and data dependent MS2 (dd-MS2) scan mode (m/z 100-1200). UPLC/QE Orbitrap MS data (.raw) were converted file format (.abf) for processing MS-Dial (http://prime.psc.riken.jp/Metabolomics_Software/MS-DIAL) to determine features, perform the alignment, and generate peak tables of m/z and the retention time for samples. Identification of RN contents based on the retention time, m/z, and the MS fragment pattern, using in public databases including Metlin (metlin-scripps.edu), the Human Metabolome Database (www.hmdb.ca) and MassBank of North America (http://mona.fiehnlab.ucdavis.edu). In addition, (+)-catechin, 4-hydroxy cinnamic acid, 5-HMF, epicatechin, glycyrrhizin, liquiritin apioside, myricetin, protocatechuic acid, and rutin were confirmed using authentic compounds

**Table S1.** List of identified compounds in roasted Nelumbinis Folium (RN).

| Compounds | Adduct | Rt(min) | Observed m/z | Fragment ion |
|---|---|---|---|---|
| 5-hydroxymethyl furfural | [M+H]+ | 1.04 | 127.0394 | 109.0283 |
| Adenosine | [M+H]+ | 0.64 | 268.1048 | 136.0627 |
| Glycyrrhizin | [M+H]+ | 7.12 | 823.4087 | 453.33698 |
| Isoquercitrin | [M+H]+ | 2.44 | 465.1037 | 85.0291 |
| Rutin | [M+H]+ | 2.06 | 611.1629 | 303.0485 |
| Trigonelline | [M+H]+ | 0.71 | 138.0555 | 94.0656 |
| Valine | [M+H]+ | 13.94 | 118.0870 | 72.0808 |
| (+)-Catechin | [M-H]- | 1.18 | 289.0724 | 245.0822 |
| 4-hydroxy cinnamic acid | [M-H]- | 2.50 | 163.0397 | 119.0492 |
| Epicatechin | [M-H]- | 1.53 | 289.0724 | 139.0393 |
| Liquiritin apioside | [M-H]- | 2.09 | 549.1635 | 135.0081 |
| Myricetin | [M-H]- | 1.68 | 317.0314 | 109.0285 |
| Protocatechuic acid | [M-H]- | 1.16 | 153.0188 | 109.0284 |
| Quercetin-4'-glucoside | [M-H]- | 2.43 | 463.0903 | 300.0305 |
| Threonic acid | [M-H]- | 0.82 | 135.0294 | 75.0071 |

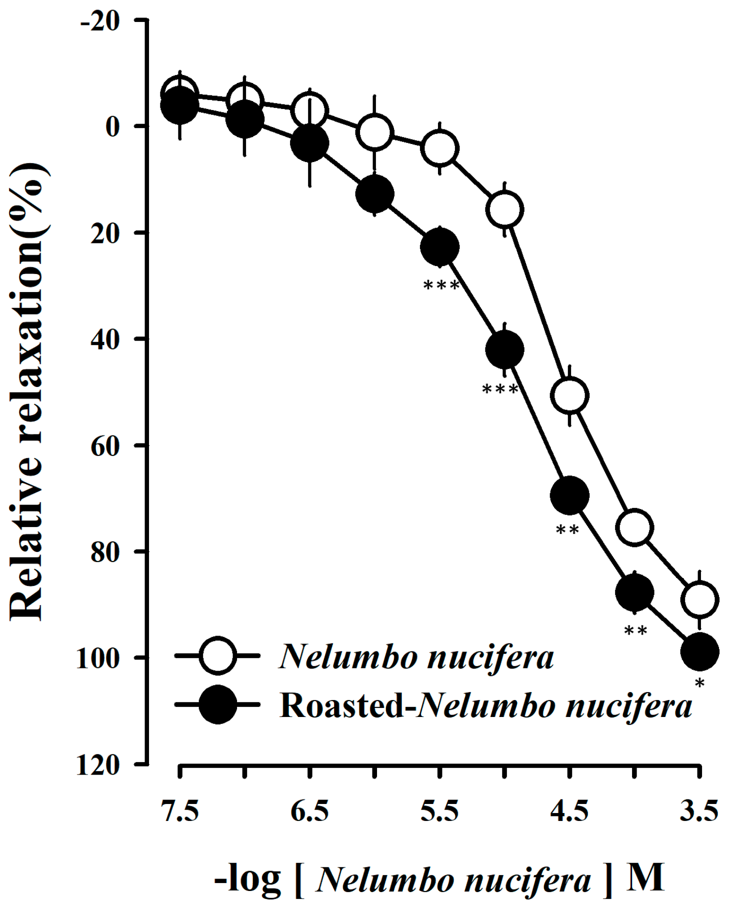


**Figure S1.** Dose-dependent vascular relaxation effect of roasted and non-roasted *Nelumbinis Folium* in thoracic aortic rings. The values represent the means ± standard error. * $p < 0.05$, and ** $p < 0.01$, and *** $p < 0.001$ vs. *Nelumbinis Folium*.
